# Supplementary material for: HIVprotI: an integrated web based platform for prediction and design of HIV proteins inhibitors
Source: J Cheminform. 2018 Mar 9;10:12. doi: 10.1186/s13321-018-0266-y (PMC5845081; doi:10.1186/s13321-018-0266-y)
Supplement: Supplementary file 2 — Additional file 2. Source code of HIVProtI web server. [file 13321_2018_266_MOESM2_ESM.zip › HIVprotI_Source-code/hivprotI/weka-3-6-6/doc/weka/core/Queue.html]

Queue


---


|  |  |  |
| --- | --- | --- |
| **Overview** \| **Package** \| **Class** \| **Tree** \| **Deprecated** \| **Index** \| **Help** \| **Weka's home** | |  |
| **PREV CLASS**   **NEXT CLASS** | **FRAMES**    **NO FRAMES**     **All Classes** |  |
| SUMMARY: NESTED \| FIELD \| CONSTR \| METHOD | DETAIL: FIELD \| CONSTR \| METHOD |  |


---


## weka.core Class Queue

```
java.lang.Object
  weka.core.Queue
```

**All Implemented Interfaces:**: java.io.Serializable, RevisionHandler

---

``` public class Queue extends java.lang.Object implements java.io.Serializable, RevisionHandler ```

Class representing a FIFO queue.

**Version:**
:   $Revision: 1.10 $

**Author:**
:   Len Trigg (trigg@cs.waikato.ac.nz)

**See Also:**: Serialized Form

---

**Constructor Summary**

`Queue()`


| **Method Summary** | |
| --- | --- |
| `boolean` | `empty()`             Checks if queue is empty. |
| `java.lang.String` | `getRevision()`             Returns the revision string. |
| `static void` | `main(java.lang.String[] argv)`             Main method for testing this class. |
| `java.lang.Object` | `peek()`             Gets object from the front of the queue. |
| `java.lang.Object` | `pop()`             Pops an object from the front of the queue. |
| `java.lang.Object` | `push(java.lang.Object item)`             Appends an object to the back of the queue. |
| `void` | `removeAllElements()`             Removes all objects from the queue m\_Tail.m\_Next. |
| `int` | `size()`             Gets queue's size. |
| `java.lang.String` | `toString()`             Produces textual description of queue. |

**Methods inherited from class java.lang.Object**

`equals, getClass, hashCode, notify, notifyAll, wait, wait, wait`

**Constructor Detail**

### Queue

```
public Queue()
```


**Method Detail**

### removeAllElements

```
public final void removeAllElements()
```

:   Removes all objects from the queue m\_Tail.m\_Next.

---


### push

```
public java.lang.Object push(java.lang.Object item)
```

:   Appends an object to the back of the queue.

    :   **Parameters:**: `item` - the object to be appended **Returns:**: the object appended

---


### pop

```
public java.lang.Object pop()
                     throws java.lang.RuntimeException
```

:   Pops an object from the front of the queue.

    :   **Returns:**: the object at the front of the queue **Throws:**: `java.lang.RuntimeException` - if the queue is empty

---


### peek

```
public java.lang.Object peek()
                      throws java.lang.RuntimeException
```

:   Gets object from the front of the queue.

    :   **Returns:**: the object at the front of the queue **Throws:**: `java.lang.RuntimeException` - if the queue is empty

---


### empty

```
public boolean empty()
```

:   Checks if queue is empty.

    :   **Returns:**: true if queue is empty

---


### size

```
public int size()
```

:   Gets queue's size.

    :   **Returns:**: size of queue

---


### toString

```
public java.lang.String toString()
```

:   Produces textual description of queue.

    :   **Overrides:**: `toString` in class `java.lang.Object`
    :   **Returns:**: textual description of queue

---


### getRevision

```
public java.lang.String getRevision()
```

:   Returns the revision string.

    :   **Specified by:**: `getRevision` in interface `RevisionHandler`
    :   **Returns:**: the revision

---


### main

```
public static void main(java.lang.String[] argv)
```

:   Main method for testing this class.

    :   **Parameters:**: `argv` - a set of strings that are pushed on a test queue


---


|  |  |  |
| --- | --- | --- |
| **Overview** \| **Package** \| **Class** \| **Tree** \| **Deprecated** \| **Index** \| **Help** \| **Weka's home** | |  |
| **PREV CLASS**   **NEXT CLASS** | **FRAMES**    **NO FRAMES**     **All Classes** |  |
| SUMMARY: NESTED \| FIELD \| CONSTR \| METHOD | DETAIL: FIELD \| CONSTR \| METHOD |  |


---
